# Conception of a Phantom in Agar-Agar Gel with the Same Bio-Impedance Properties as Human Quadriceps

**DOI:** 10.3390/s21155195

**Published:** 2021-07-31

**Authors:** Margaux Peixoto, Marie-Valérie Moreno, Nassim Khider

**Affiliations:** 1 Research Center, RunSys, 53 Avenue Carnot, 69250 Neuville-sur-Saône, France; peixoto.margaux@gmail.com; 2 C-19, 19 Cours Blaise Pascal, 91000 Évry-Courcouronnes, France; nassim.khider@c-19.fr; 3 ENSIIE, 1 Rue de la Résistance, 91000 Évry-Courcouronnes, France

**Keywords:** phantom, bioimpedance, agar-agar, NaCl, graphite, muscle

## Abstract

The physiology of the patient can be reflected by various data. Serious games, using an intelligent combination, could be based on this data to adjust to the specificities of the patient. Rehabilitation would therefore be personalized to the patient. This smart suit would use dry electrodes in order to be easily usable. Before performing dry electrode validation tests on a population, it is necessary to perform preliminary tests on a phantom. Agar-Agar (AA) gel, combined with NaCl and graphite which directly impact the resistivity and reactance values of the phantom, are generally used. Depending on the part of the body simulated by the phantom, it is necessary to adapt the concentrations of NaCl and graphite in order to obtain values of physiological reactance and resistance. The anisotropy of a muscle must also be considered. Different concentrations of NaCl and graphite have been tested in order to present charts linking the concentrations to the resistance and reactance values of the AA phantom. Electrical properties similar to those of human quadriceps are achieved at a concentration of 7 g/L of NaCl and 60 g/L of graphite. These values can be used as a conversion table to develop an AA phantom with electrical properties similar to different muscles. Furthermore, an AA phantom has an anisotropy of 0° and 90°. This anisotropy corresponds to a human quadriceps, where 0° is the direction of the muscle fiber. This will allow us to study and characterize the behavior of the electrodes on an anisotropic model. Thus it can be used as a first test phase for dry electrodes in order to propose the most suitable conditions for a connected garment application.

## 1. Introduction

The first ‘serious game’, according to the contemporary definition (a playful simulation for serious learning), appeared at the beginning of the 19th century. At that time, these games were used to train the military in various war tactics. It was not until the end of the 20th century, and the work of Abt [1] entitled *Serious Games*, that serious games were popularized to include educational, political, and marketing training via board games, outdoor games and, finally, computer games. At the beginning of the 2000s, the field developed further, notably as a result of numerous academic works, the classification of *Serious Games*, and the creation of databases by Alvarez and Djaouti [2]. The health sector is gradually taking over these solutions, bringing a playful dimension to an often difficult world. In this context, these serious games can then have various objectives [3,4,5,6,7,8]: preparing a patient for an operation, helping the patient manage a chronic disease, enabling rehabilitation, training staff, etc. During this same period, also due to the emergence of computer solutions, the first virtual reality devices were born, with Daniel Vickers’ helmet created in 1970 at the University of Utah. The field then continued to develop through various research studies and companies offering devices that were increasingly accessible to the general public (VPL Research (1985), Sega (1990), Apple (1994), Google (2007), etc.) [9,10,11,12,13]. The evolution of computing, communications (smartphones, etc.), and electronics (lower costs, greater efficiency, reduced space requirements, etc.) have enabled biomedical engineering to develop and amplify the emergence of health and wellness services based on increasingly ergonomic and embedded sensors (ECG bracelets, smart clothing, etc.) using reusable, washable, dry (no gel) electrodes [14,15].

These biomedical measurement systems produce a lot of data (Big Data). These data can be used to adapt the game to the specificities of each individual. This also allows the evolution of the patient’s physiological data to be monitored and thus to improve his or her rehabilitation. 

The ϕ-Game project is part of the field of serious games. It aims to offer a solution based on virtual or augmented reality, and using an ecosystem of electrophysiological sensors, to health and sports professionals, as well as to the general public. Muscles are strongly anisotropic, and their electrical properties are altered during contractions [16,17,18]. A local impedance measurement can therefore identify a muscle contraction. This can be valuable in the context of a monitoring rehabilitation or for applications in games or serious games. Indeed, following a muscle contraction in real time allows the game to be adapted to the patient’s condition (speed and/or intensity of the effort to be provided according to the patient’s muscular fatigue, for example). This project focuses on bioelectrical impedance analysis (BIA), but the use of a smart suit for a serious game application will be able to use various physiological signals as appropriate. 

In order to create a smart suit implemented with this technology, the choice of the electrode implemented is crucial. Bioimpedance measurements are generally measured using gel electrodes. However, for a smart suit application, the electrode must be an integral part of the garment. It must therefore be reusable and washable, and must reliably and accurately acquire the body’s electrical signals [19,20,21]. Different textile electrodes are available. In order to make a relevant material choice, it is necessary to characterize and compare the different electrodes available [22,23]. Before testing on human subjects, a preliminary phantom test step is necessary.

The first step of this project focuses on the study of the contraction of the quadriceps. The aim of this study is therefore to define the composition of a phantom with electrical characteristics close to those of the human quadriceps. Phantoms in agar-agar gel, hereinafter referred to as AA phantom, simulating the electrical properties of the human body have already been carried out, however, these correspond to the properties of the whole body and are not specific to a muscle [24,25]. 

It is possible to influence the resistance (R in Ω) and reactance (X in Ω) by changing the NaCl and graphite concentrations of the AA phantom. The optimal NaCl and graphite concentrations will be determined as well as the weight to be placed on the electrodes to obtain a satisfactory contact impedance. This AA phantom can then be used to test the contact impedance of different dry electrodes and can be reused for further studies on the quadriceps. 

The module used for the tests allows bipolar and quadripolar measurements. Bipolar measurements provide information on the contact interface, while quadripolar measurements are used to characterize the electrical properties of the gel. The notion of bioimpedance, noted as ’Z’ will also be used. Z is defined such that Equation (1):

(1) $$Z=\sqrt{R^{2}+X^{2}}$$

where impedance (Z in Ω), resistance (R in Ω) and reactance (X in Ω).

**Figure 1:**
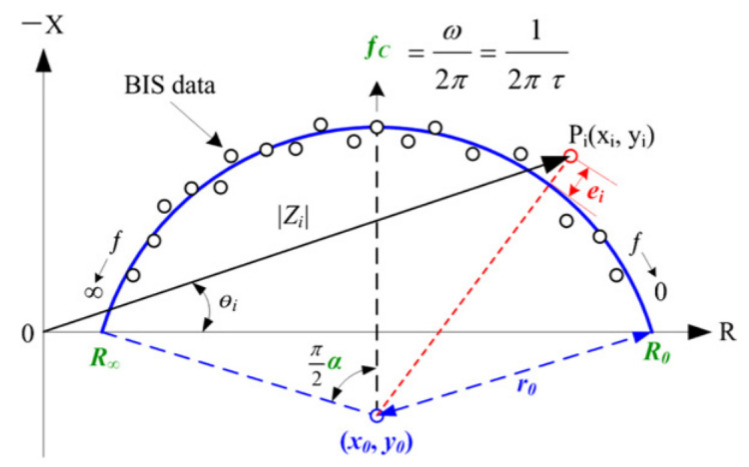
Cole–Cole curve model [26].

The evolution of (R) and (X) of an AA phantom will be compared to the model proposed by Cole–Cole [26]. This model allows the characterization of different parameters of biological tissues with electrical data, as shown in Figure 1. 

The objective of this study is to design an agar-agar gel quadriceps phantom with electrical properties close to those of a human quadriceps. This phantom will then be used to carry out the first characterization tests on textile electrodes before being tested on human subjects. Conversion tables allowing a phantom with different resistance and reactance values will be created.

## 2. Materials and Methods

### 2.1. AA Phantom Preparation Protocol

The reagents and materials used in the AA phantom development protocol are detailed in Table 1 and Table 2. 

AA phantom preparation protocol: 

*a*: weight of NaCl (g); *b*: weight of graphite (g);

- Wash all utensils with NaClO,
- Dose *a* g of NaCl,
- Dose 4 g of agar-agar,
- Dose *b* g graphite,
- Dose 1 L of demineralized water,
- Mix the demineralized water and NaCl in the pan, bring to a boil and then stop quickly in order to limit evaporation losses. This mixture is then referred to as salt water.
- Dose 100 mL of salt water,
- Place the salt water in the pan, quickly add the agar-agar and graphite while stirring, then increase the heat gradually until bubbles appear at the bottom of the pan,
- Quickly place into the plastic box and put in the refrigerator (0.8 °C) for at least six hours.

Quantities *a* and *b* will be determined next.

AA phantom utilization protocol:

- Take the AA phantom out of the box, place it upside down on a non-conductive surface and leave it at ambient temperature for one hour,
- If the AA phantom is wet, wipe it with a tissue,
- Positioning the electrodes,
- Place a 250 g weight on the electrodes,
- Measure,
- Put the AA phantom in the box and put it in the refrigerator.

### 2.2. Materials

Table 3 shows the acquisition module used during the tests as well as the used electrodes.

**Figure 2:**
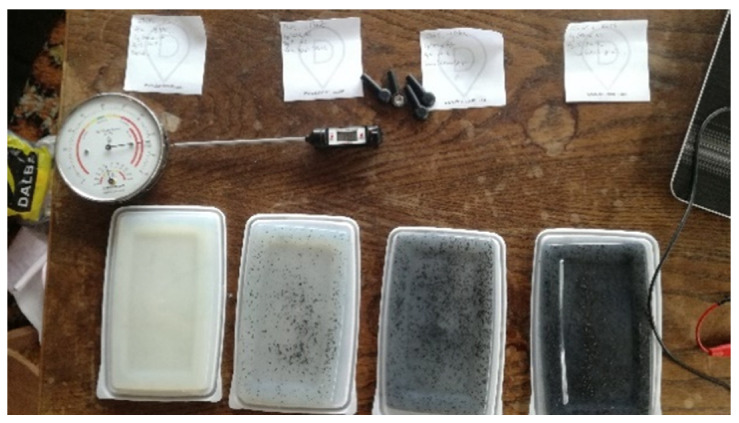
Gradient of 4 AA phantom of different graphite concentration (0, 12, 24 and 36 g/L).

**Figure 3:**
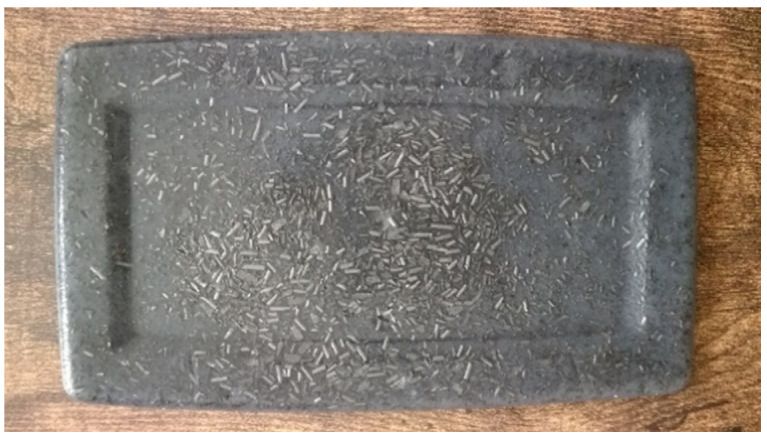
AA phantom simulating a human quadriceps (NaCl concentration = 7 g/L; graphite concentration = 60 g/L).

Figure 2 shows the AA phantoms with different graphite concentrations. The color gradient due to the difference in concentration can be seen. The AA phantom simulating a human quadriceps is shown in Figure 3.

### 2.3. Resistance and Reactance Acquisition Method

**Figure 4:**
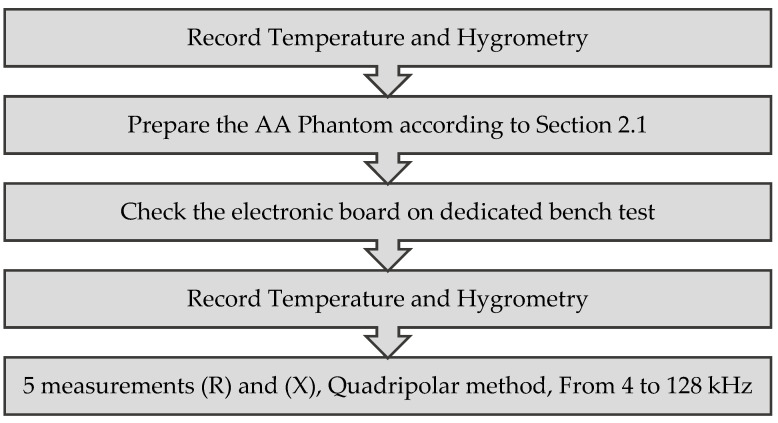
Test procedure and workflow diagram.

For each AA phantom, five measurements of resistance (R) and reactance (X) (in quadripolar covering frequencies from 4 to 128 kHz) are taken in order to verify the repeatability of the measurement. This approach will be used for all (R) and (X) measurements. During the preparation of the AA phantoms and then during the tests, the temperature and hygrometry of the environment are recorded. Before each series of tests, the electronic board is verified using an electrical phantom of known resistance and reactance. Before each series of tests, checks are carried out to ensure the correct operating module, with using an electrical phantom of known resistance and reactance. The different steps of the test procedure are explained in Figure 4.

### 2.4. Determination of NaCl Concentration for Resistance

Several studies have shown that the addition of NaCl increases the conductivity of the AA phantom [26,27,28]. The NaCl concentration must be determined to match the resistance values in the literature [17,18]. The different concentrations tested are 0 and from 1 to 7 g/L. An NaCl-free AA phantom composed only of agar-agar is also prepared as a control. 

Temperature and hygrometry are controlled. The temperature during the preparation of the AA phantom and then during the tests was 23.1 °C and 22.5 °C (+/−0.1 °C) respectively. The humidity during the preparation of the AA phantom and during the tests was 52.0% and 51.0 % (+/−0.5%) respectively.

### 2.5. Determination of Graphite Concentration for Reactance

Graphite mainly influences the electrical reactance of the AA phantom [25,28]. Once the NaCl concentration is fixed, the graphite concentration is determined to correspond to the reactance values of a human quadriceps in the literature [17,18]. The concentrations tested were: 0, 12, 24, 36, 48, 60 g/L (±1 g/L). A control AA phantom of identical NaCl concentration but without graphite is prepared.

Temperature and hygrometry have been controlled. The temperature during the preparation of the AA phantom and then during the tests was 24.3 °C and 22.6 °C (+/−0.1 °C) respectively.

The humidity during the preparation of the AA phantom and during the tests was 54.0 % and 50.0 % (+/−0.5%) respectively.

### 2.6. Determination of the Weight Placed on the Electrodes to Obtain a Good Contact at the Interface

In order to provide a good contact at the interface, it is necessary to apply pressure to the electrodes [20]. A container is placed (mass = 32 g; weight = 0.26 g/cm²) on the AA phantom. Mass is gradually increased (in increments of 20 g) until a total mass of 250 g is obtained on the AA phantom (corresponding to a weight of 2 g/cm²). The appearance of the AA phantom is visually monitored to make sure that the addition of the weight does not deteriorate it. 

Reactance (X) is measured in bipolar mode (tests are carried out at a low frequency (4) to be in the most unfavorable conditions in terms of contact impedance), without weight, after addition of the container and then with each addition of mass. 

### 2.7. AA Phantom Anisotropy

Human muscles are highly anisotropic. This anisotropy has already been characterized, especially on meat [29,30]. To characterize the anisotropy of the phantom, three bipolar measurements of (R) and (X) are taken at 0°, 30°, 60° and 90° [30] with 0° corresponding to the width of the phantom. The electrodes are placed at a distance of 4 cm. Temperature and hygrometry are controlled, respectively, at 25.9 °C and 38.0%.

### 2.8. AA Phantom Stability over Time

Once the NaCl and graphite concentrations have been determined, the final AA phantom is measured on days 1, 2, 3, 4 and 5 in order to study the temporal stability of the AA phantom on the values of (X) and (R). According to the literature, it can be stored for several days in the refrigerator. Between each measurement, the AA phantom is kept in the refrigerator. The visual aspect of the AA phantom is also controlled by the investigator, as are temperature and hygrometry (Table 4).

## 3. Results

### 3.1. NaCl Concentration

**Figure 5:**
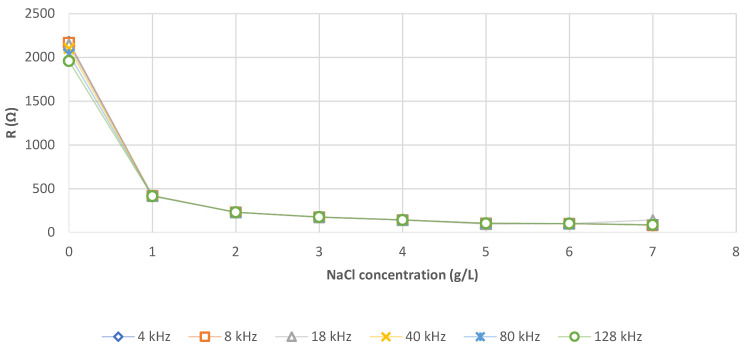
Evolution of resistance (R in Ω) as a function of the NaCl concentration of the AA phantom for frequencies ranging from 4 kHz to 128 kHz (Table A1).

As expected, Figure 5 shows that when the NaCl concentration increases, the resistance decreases. 

The resistance values obtained do not correspond to human physiology, between 0 and 1 g/L of NaCl. For 1 g/L, the values correspond to the whole body and for 7 g/L to those of the quadriceps. An NaCl concentration of 7 g/L is then chosen. Graphically, the frequency of acquisition does not seem to have an impact, except between 0 and 1 g/L of NaCl. However, we do obtain mathematically significant difference *p* < 0.001 (Shapiro–Wilk test *p* < 0.001, then a non-parametric Kruskal–Wallis test).

Depending on the expected R (x), Equations (2)–(7) are used to determine the required NaCl (y) concentration (Table 5). 

### 3.2. Graphite Concentration

**Figure 6:**
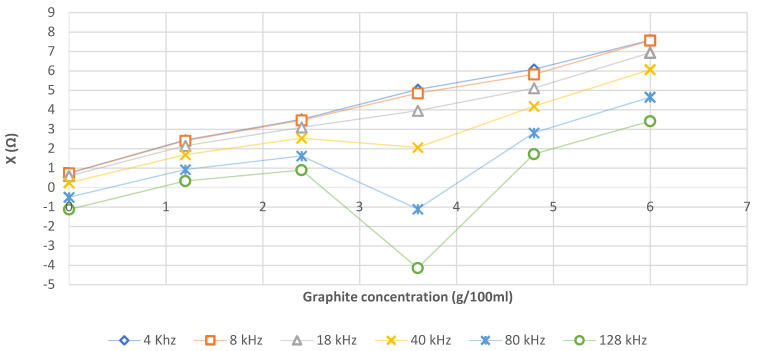
Evolution of reactance (X in Ω) as a function of the graphite concentration of the AA phantom for frequencies ranging from 4 kHz to 128 kHz.

Figure 6 shows that the addition of graphite increases the reactance X for frequencies ranging from 4 to 18 kHz and this increase is linear.

For 60 g/L of the mixture of salt water and graphite, X up to 7 Ω, which corresponds to the literature values [17,18]. 

Depending on the expected reactance (X) (x) and the frequency, the Equations (8)–(13) presented in Table 6 are used to determine the required graphite concentration (y). Due to aberrant values at 36 g/L of graphite, regressions are established without these points for 40, 80 and 128 kHz. It may be considered that this specific concentration generates self-inductance effects that increase with frequency, and which cancel each other out for a higher concentration.

**Figure 7:**
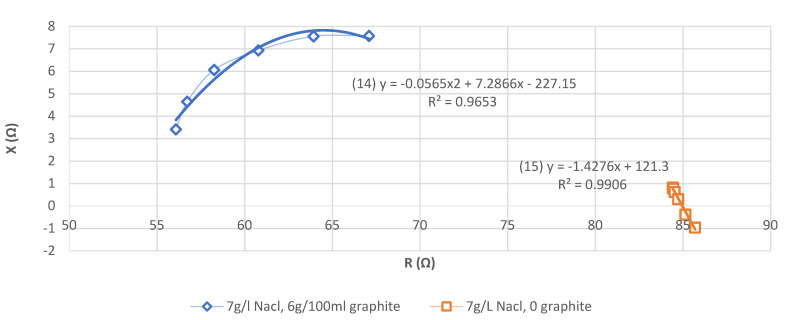
Comparison of the evolution of (R) versus (X) (Cole–Cole curve) for an AA phantom with and without the addition of graphite.

Figure 7 shows that for the AA phantom without graphite, the evolution of (X) is linear. It is an AA phantom simulating a resistive AA phantom without any reactive properties, rather than a muscle [19].

For the AA phantom with graphite, the evolution of (R) and (X) approaches the Cole–Cole [27] curve found in the literature [28] for the physiology of human skeletal muscle. The addition of graphite increases (X).

### 3.3. Weight Applied over the Electrodes 

**Figure 8:**
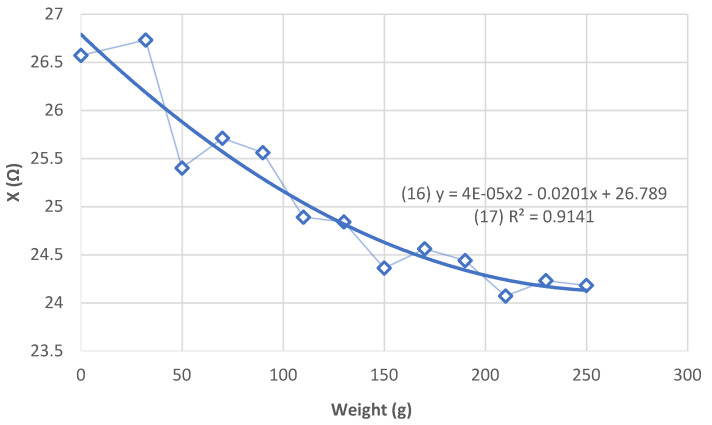
Evolution of X (Ω) depending on the weight applied to the electrodes (Table A2).

X (corresponding at the contact reactance measured in the bipolar method) decreases with increasing weight (Figure 8).

For 250 g, the value of (X) is constant. The addition of additional mass is not necessary and could damage the AA phantom. A 250 g weight is subsequently placed on the electrodes. 

### 3.4. AA Phantom Anisotropy 

The values of (R), (X) and (Z) depending on the angle of the AA phantom were tested and listed in Table 7.

**Figure 9:**
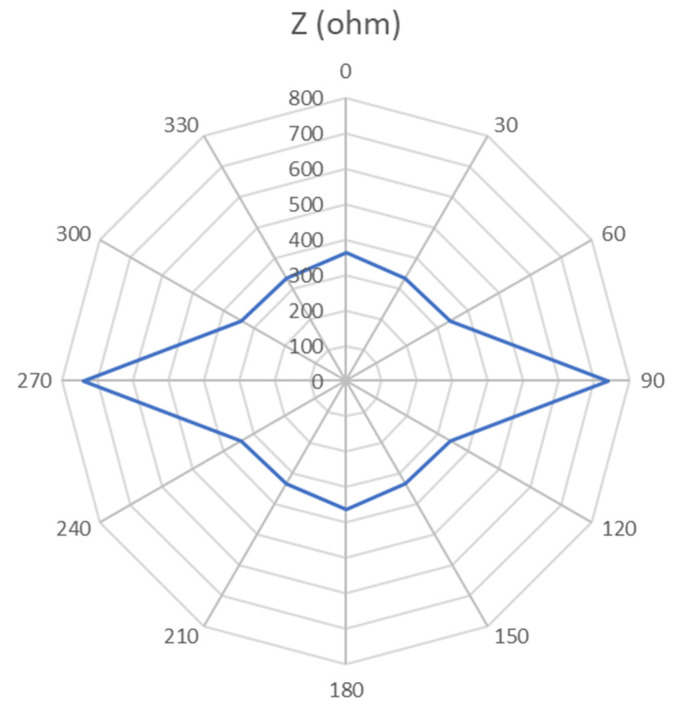
Polar diagram of the evolution of (Z) (Ω) as a function of the measurement angle.

Figure 9 shows the anisotropy of the AA phantom on a polar diagram. The anisotropy of the AA phantom at 90° is similar to the literature for some types of muscles [30]. In contrast, it is not found at 30° and 60°. 

### 3.5. Stability over Time

**Figure 10:**
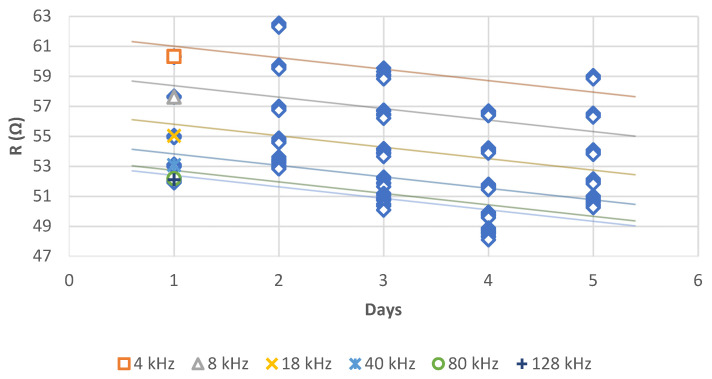
Regression of (R) (Ω) per days (*p* < 0.001 *** for 4, 8, 18 and 40 kHz; *p* > 0.05 for 80 and 128 kHz; with R² equal respectively to 0.402, 0.389, 0.372, 0.377, 0.416, 0.471) (Table A3).

Time has a significant impact on (R) (Figure 10). It decreases linearly over time. Low frequencies have an impact on (R). However, above 80 kHz, the frequency factor no longer has a significant impact on (R).

**Figure 11:**
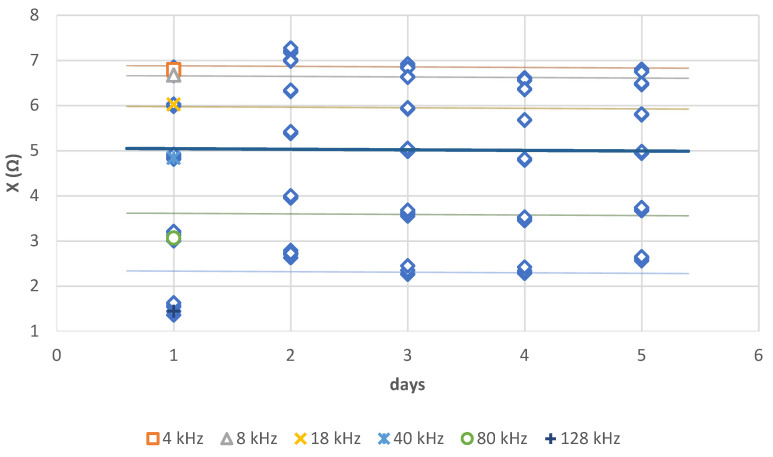
Regression of (X) (Ω) per days (*p* > 0.05, with R² equal respectively to 0.256, 0.519, 0.490, 0.127, 0.068, 0.301) (Table A3).

Contrary to (R), time does not have a significant impact on (X) (Figure 11). On the other hand, low frequencies have an impact on (X) in a similar way to (R). Moreover, above 80 kHz, frequency no longer has an impact on the value of (X). 

Subsequently, all the measurements on the AA phantoms will be carried out on D1 to avoid any bias due to the evolution in time of the AA phantom.

## 4. Discussion

For NaCl concentration, we noticed that under a concentration of 1g/L, the phantom is too electrically insulating to allow any available value. As expected, then there is an inverse correlation between the concentration of NaCl ions and the resistance (R) due to the increase of the conductivity of the phantom. As R is not a function of the frequency, we notice logically a non-significant difference between the curve obtained for each frequency. We propose different equations for each frequency which could be replaced by one multifrequency equation.

For graphite concentration, as expected, we noticed a correlation between the graphite concentration and the value of the reactance (X). As X is a function of the frequency, we obtained logically a significative difference between the curve obtained for each frequency. 

Even if it is necessary during the preparation of the phantom to distinguish R and X according to the frequency, we verified that our phantom verifies the Cole–Cole curve characteristic, used commonly in bioimpedance field. 

We noticed a significantly inverse correlation between the weight put on the electrode and the reactance due to the decrease of the contact impedance. After 250 g, the contact impedance is stable, then we do not observe evolution. Worst we risk is damage to the phantom. 

Thanks to the terrestrial electromagnetic field, the graphite bars orient towards magnetic north simulating an anisotropy between longitudinal and transverse features. Unfortunately, we did not find a method to obtain more anisotropic angles. 

We hypothesized that time as a significant inverse effect on resistance due to the evaporation on water in fridge that generated a thin layer of water decreased the resistance even in quadripolar method. In contrary, time has no effect on reactance because the graphite bars do not move and are not impacted by the fridge.

For NaCl and graphite concentration, the values chosen for the quadriceps AA phantom were not exceeded in the conversion table. It would be interesting to test 9 g/L of NaCl as well as 72 g/L of graphite. It would also be interesting to test the NaCl concentration between 0 and 1 g at a shorter interval (every 0.1 g). The concentration of NaCl and graphite has an impact on both resistance and reactance. It is therefore possible that another concentration combination works for a quadriceps phantom. Nevertheless, we obtain an AA phantom with electrical properties close to those of a human quadriceps [17,18]. 

Monitoring the addition of weight does not allow a value to be defined from which the contact impedance is available. We chose a high value to ensure a low impedance while ensuring that the addition of the weight did not damage the AA phantom.

The suggested AA phantom presents an anisotropy close to that observed on meat [29,30] between 0° and 90°. This anisotropy comes from the orientation of graphite particles under the effect of the earth’s electromagnetic field. Other authors could thus observe a different anisotropy depending on their locations of experimentation. On the other hand, the layer of skin present on the muscle is not reproduced by the AA phantom. Different concentrations of agar-agar, NaCl and graphite could improve the phantom by adding a layer with electrical properties similar to that of the skin.

Only stability over time of the AA phantom was tested over several days. The variation in (R) observed during the stability study of the AA phantom over time can be explained by a variation in external conditions (temperature and humidity). Indeed, it is possible that the phantom may dry out if humidity and temperature are not controlled. This variation in humidity has a direct impact on the value of (R). On the other hand, time does not have an impact on (X). Indeed, the graphite is caught in the agar-agar gel. Its concentration therefore does not vary over time. Moreover, the frequency impacts (R) and (X) below 80 kHz. Indeed, at low frequencies, the current does not pass through all the AA phantom. There is a gradient of NaCl and graphite due to gravity. For high frequencies, the current passes through the entire gel. The frequency no longer has an impact upon (R) and (X). The measurements were always performed after leaving the AA phantom in ambient conditions for one hour. It could be interesting to study the ageing of the AA phantom in the open air. Finally, if the AA phantom is used to characterize material, such as sensors or electrodes, it is possible to keep the AA phantom for several days as long as its characteristics are determined before each series of tests.

Finally, the temperature and hygrometry of the environment during the preparation of the AA phantoms and then during the tests were recorded. However, an advanced study of the impact of these factors on the AA phantoms would allow us to define the conditions of use. We nevertheless managed to obtain a repeatable protocol without controlling these parameters and staying within the average value ranges of the inside of a building.

It would be interesting to precisely control the experimental conditions of temperature and hygrometry in order to study their impact on the AA phantoms. 

## 5. Conclusions

We studied how to determine the concentration of NaCl and graphite to obtain the expected electrical characteristics in the agar-agar phantom simulating a human quadriceps. These properties are obtained for a NaCl concentration of 7 g/L and 60 g/L of graphite salt solution.

At first, this AA phantom will be used to test different textile electrodes for connected garment applications. This inexpensive solution could, in the future, be used to carry out preliminary studies before carrying them out on human subjects. The anisotropy due to the orientation of the muscle fibers is reproduced. A study of the stability of the AA phantom over time has also been carried out. It is preferable to use the AA phantom the day after its preparation. It is possible to reuse the phantom up to five days after its preparation, but its properties may have changed. It is therefore necessary to determine these properties before testing. This document also provides a conversion table. It allows to choose the concentrations of NaCl and graphite according to the desired electrical properties of the AA phantom.

This study can also be used for future work to improve muscle phantom models. Indeed, the skin layer covering the muscle could be added. In addition, simulating different angles of anisotropy would allow for different muscle connections to be added to the proposed model.

## Figures and Tables

**Table 1:** Reagents necessary for the preparation of the AA phantom.

Reagents	References
Demineralized water	Brand: Mieuxa; conductivity (20 °C) <10 µS/cm
NaCl	Baleine brand; salt, sea salt, potassium fluoride: 250 mg/100 g, sodium iodide: 15 to 20 mg/kg, anti-caking agents: magnesium carbonates, e530, e535
Graphite	BIC brand: HB 0.7 mm pencil lead
Natural Gelling Agar-Agar	Vahiné Brand, vegetal gelling; composition: agar E406

**Table 2:** Materials necessary for development of the AA phantom.

Equipment	References
Graduated cylinder 250 mL	+/– 1 mL
Precise scale	Capacity 300 g; +/–0.1 g
Pan	
Plastic box 175 × 110 × 33 mm	
Tablespoon	
NaClO	

**Table 3:** Materials used for AA phantom testing.

Equipment	References/Characteristics	
An electronic board of bioimpedance (Module Phi-Light, RunSys, France)	Bipolar/Quadripolar ->from 10 *V/V* to 80 *V/V* -> from 8 to 96 μA -> from 125 Hz to 300 kHz, -> Accuracy on R//RC bench test: R 0.006 ± 0.005 Ω X 0.003 ± 0.011 Ω -> Resolution on R//RC bench test: R 0.0008 ± 0.0008 Ω X 0.009 ± 0.007 Ω	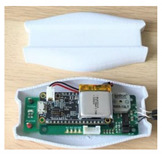
Repositionable monitoring electrodes 3M Red Dot electrodes Ag/AgCl gel	ref 2660-5	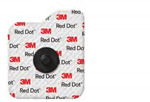

**Table 4:** Temperature and humidity conditions during the AA phantom time stability test.

	D1	D2	D3	D4	D5
**Temperature (°C) +/−0.1**	22.8	22.3	23.5	22.3	22.7
**Humidity (%) +/−0.5**	58.0	46.0	48.0	49.0	60.0

**Table 5:** Polynomial regression between NaCl concentration (y) and x (Ω); the resistance wanted depending on the frequency.

Frequencies (kHz)	y (NaCl Concentration)	R²
4	(2) y = 1523.6x^−1.213^	0.9935
8	(3) y = 1531.1x^−1.214^	0.9935
18	(4) y = 1857.1x^−1.237^	0.8599
40	(5) y = 1570.6x^−1.219^	0.9934
80	(6) y = 1616.9x^−1.224^	0.9932
128	(7) y = 1670x^−1.23^	0.9931

**Table 6:** Linear regression between graphite concentration (y) to find and x (Ω) the reactance (X) wanted depending on the frequency.

Frequencies (kHz)	y (Graphite Concentration)	R²
4	(8) y = 0.898x − 0.7968	0.9964
8	(9) y = 1.088x + 0.8722	0.9942
18	(10) y = 0.999x − 0.6335	0.9852
40	(11) y = 1.094x − 0.3351	0.9794
80	(12) y = 1.094x − 0.3351	0.9794
128	(13) y = 1.433x + 1.3856	0.9376

**Table 7:** Evolution of (R), (X) and (Z) as a function of the electrode placement angle.

Angle (°)	R (Ω)	X (Ω)	Z (Ω)
0	360.56	28.93	361.72
30	335.09	23.22	335.89
60	338.01	27.99	339.17
90	739.35	49.00	740.97
120	338.01	27.99	339.17
150	335.09	23.22	335.89
180	360.56	28.93	361.72
210	335.09	23.22	335.89
240	338.01	27.99	339.17
270	739.35	49.00	740.97
300	338.01	27.99	339.17
330	335.09	23.22	335.89

## Data Availability

Data available in a publicly accessible repository that does not issue DOIs. You find them in those following Appendix A.

## Appendix A

**Table A1:** Raw data: NaCl concentration.

NaCl Concentration (g/L)	Frequency (kHz)	R Mean (Ω)
0	4	2174.648
1	4	419.482
2	4	229.996
3	4	174.182
4	4	141.266
5	4	102.882
6	4	99.834
7	4	84.428
0	8	2163.974
1	8	418.9
2	8	229.912
3	8	174.186
4	8	141.266
5	8	102.87
6	8	99.82
7	8	84.456
0	18	2142.62
1	18	418.152
2	18	229.822
3	18	174.246
4	18	141.314
5	18	95.1285
6	18	99.862
7	18	141.314
0	40	2101.654
1	40	417.018
2	40	229.744
3	40	174.434
4	40	141.49
5	40	103.004
6	40	99.936
7	40	84.736
0	80	2035.366
1	80	415.618
2	80	229.8
3	80	174.918
4	80	141.912
5	80	103.258
6	80	100.214
7	80	85.156
0	128	1957.778
1	128	414.578
2	128	230.044
3	128	175.6
4	128	142.506
5	128	103.626
6	128	100.602
7	128	85.698

**Table A2:** Raw data: Evolution of X depending on the weight applied on the electrodes for a frequency range from 4 kHz to 128 kHz.

Weight (g)	Frequency kHz	X (Ω)
0	4	26.57
	8	24.48
	18	21.17
	40	18.66
	80	16.44
	128	15.74
32	4	26.73
	8	24.19
	18	20.87
	40	18.17
	80	15.83
	128	14.94
50	4	25.4
	8	23.45
	18	20.27
	40	17.76
	80	15.43
	128	14.34
70	4	25.71
	8	23.25
	18	20.09
	40	17.6
	80	15.16
	128	14.11
90	4	25.56
	8	22.95
	18	19.83
	40	17.26
	80	14.73
	128	13.66
110	4	24.89
	8	22.84
	18	19.69
	40	17.12
	80	14.58
	128	13.48
130	4	24.84
	8	22.67
	18	19.51
	40	16.93
	80	14.42
	128	13.24
150	4	24.36
	8	22.45
	18	19.35
	40	16.79
	80	14.32
	128	13.09
170	4	24.56
	8	22.3
	18	19.25
	40	16.69
	80	14.19
	128	12.92
190	4	24.44
	8	22.17
	18	19.14
	40	16.6
	80	14.07
	128	12.85
210	4	24.07
	8	22.21
	18	19.14
	40	16.58
	80	14.06
	128	12.85
230	4	24.23
	8	22.16
	18	19.04
	40	16.49
	80	13.92
	128	12.7
250	4	24.18
	8	22.12
	18	19.02
	40	16.44
	80	13.89
	128	12.61

**Table A3:** Raw data: Evolution of R and X over time (during 5 days) for a frequency range from 4 kHz to 128k Hz.

Days	Frequency kHz	R (Ω)	X (Ω)
1	4	60.35	6.84
1	4	60.31	6.79
1	4	60.28	6.81
1	4	60.28	6.85
1	4	60.26	6.82
2	4	62.53	7.17
2	4	62.47	7.18
2	4	62.40	7.17
2	4	62.32	7.22
2	4	62.27	7.27
3	4	59.54	6.91
3	4	59.31	6.91
3	4	59.11	6.87
3	4	59.01	6.92
3	4	58.82	6.83
4	4	56.68	6.61
4	4	56.64	6.56
4	4	56.56	6.55
4	4	56.49	6.61
4	4	56.37	6.58
5	4	59.04	6.80
5	4	59.00	6.80
5	4	58.95	6.74
5	4	58.88	6.75
5	4	58.82	6.74
1	8	57.67	6.66
1	8	57.61	6.67
1	8	57.63	6.72
1	8	57.59	6.67
1	8	57.59	6.73
2	8	59.74	6.98
2	8	59.66	6.98
2	8	59.59	6.99
2	8	59.52	7.01
2	8	59.48	6.99
3	8	56.74	6.64
3	8	56.68	6.64
3	8	56.48	6.64
3	8	56.40	6.62
3	8	56.20	6.63
4	8	54.22	6.35
4	8	54.14	6.37
4	8	54.05	6.36
4	8	53.99	6.36
4	8	53.87	6.36
5	8	56.53	6.46
5	8	56.48	6.46
5	8	56.44	6.47
5	8	56.35	6.49
5	8	56.28	6.49
1	18	55.05	5.98
1	18	55.02	6.02
1	18	54.99	6.00
1	18	54.98	5.99
1	18	54.91	6.03
2	18	56.99	6.31
2	18	56.88	6.31
2	18	56.82	6.31
2	18	56.76	6.34
2	18	56.72	6.34
3	18	54.17	5.92
3	18	54.08	5.93
3	18	53.92	5.93
3	18	53.84	5.92
3	18	53.65	5.95
4	18	51.8	5.67
4	18	51.71	5.68
4	18	51.63	5.68
4	18	51.57	5.68
4	18	51.44	5.68
5	18	54.09	5.79
5	18	54.03	5.80
5	18	53.95	5.80
5	18	53.87	5.81
5	18	53.79	5.81
1	40	53.13	4.81
1	40	53.07	4.84
1	40	53.05	4.88
1	40	53.00	4.92
1	40	52.96	4.92
2	40	54.87	5.38
2	40	54.74	5.41
2	40	54.67	5.42
2	40	54.61	5.42
2	40	54.56	5.42
3	40	52.27	4.98
3	40	52.14	5.01
3	40	51.9	5.01
3	40	51.85	5.04
3	40	51.64	5.05
4	40	49.93	4.79
4	40	49.83	4.79
4	40	49.77	4.81
4	40	49.7	4.82
4	40	49.57	4.82
5	40	52.16	4.94
5	40	52.08	4.96
5	40	51.99	4.98
5	40	51.92	4.98
5	40	51.83	4.98
1	80	52.26	3.00
1	80	52.17	3.06
1	80	52.1	3.13
1	80	52.03	3.19
1	80	52.01	3.21
2	80	53.62	3.95
2	80	53.47	3.97
2	80	53.4	3.99
2	80	53.35	4.00
2	80	53.3	4.00
3	80	51.19	3.55
3	80	51.05	3.59
3	80	50.81	3.62
3	80	50.72	3.64
3	80	50.48	3.68
4	80	48.89	3.45
4	80	48.81	3.49
4	80	48.72	3.50
4	80	48.63	3.51
4	80	48.5	3.53
5	80	51.03	3.67
5	80	50.94	3.7
5	80	50.85	3.71
5	80	50.76	3.74
5	80	50.67	3.74
1	128	52.21	1.36
1	128	52.09	1.44
1	128	51.99	1.54
1	128	51.91	1.57
1	128	51.9	1.63
2	128	53.22	2.63
2	128	53.06	2.70
2	128	52.95	2.74
2	128	52.85	2.79
2	128	52.83	2.73
3	128	50.87	2.26
3	128	50.73	2.27
3	128	50.49	2.35
3	128	50.35	2.45
3	128	50.08	2.45
4	128	48.57	2.28
4	128	48.52	2.29
4	128	48.34	2.34
4	128	48.29	2.33
4	128	48.10	2.42
5	128	50.6	2.56
5	128	50.47	2.62
5	128	50.43	2.61
5	128	50.36	2.62
5	128	50.25	2.65
